# Application of Large-Scale Parentage Analysis for Investigating Natal Dispersal in Highly Vagile Vertebrates: A Case Study of American Black Bears (*Ursus americanus*)

**DOI:** 10.1371/journal.pone.0091168

**Published:** 2014-03-12

**Authors:** Jennifer A. Moore, Hope M. Draheim, Dwayne Etter, Scott Winterstein, Kim T. Scribner

**Affiliations:** 1 Biology Department, Grand Valley State University, Allendale, Michigan, United States of America; 2 Department of Zoology, Michigan State University, East Lansing, Michigan, United States of America; 3 Wildlife Division, Michigan Department of Natural Resources, East Lansing, Michigan, United States of America; 4 Department of Fisheries and Wildlife, Michigan State University, East Lansing, Michigan, United States of America; Instituto de Higiene e Medicina Tropical, Portugal

## Abstract

Understanding the factors that affect dispersal is a fundamental question in ecology and conservation biology, particularly as populations are faced with increasing anthropogenic impacts. Here we collected georeferenced genetic samples (n = 2,540) from three generations of black bears (*Ursus americanus*) harvested in a large (47,739 km^2^), geographically isolated population and used parentage analysis to identify mother-offspring dyads (n = 337). We quantified the effects of sex, age, habitat type and suitability, and local harvest density at the natal and settlement sites on the probability of natal dispersal, and on dispersal distances. Dispersal was male-biased (76% of males dispersed) but a small proportion (21%) of females also dispersed, and female dispersal distances (mean ± SE  =  48.9±7.7 km) were comparable to male dispersal distances (59.0±3.2 km). Dispersal probabilities and dispersal distances were greatest for bears in areas with high habitat suitability and low harvest density. The inverse relationship between dispersal and harvest density in black bears suggests that 1) intensive harvest promotes restricted dispersal, or 2) high black bear population density decreases the propensity to disperse. Multigenerational genetic data collected over large landscape scales can be a powerful means of characterizing dispersal patterns and causal associations with demographic and landscape features in wild populations of elusive and wide-ranging species.

## Introduction

Dispersal is an important ecological process that allows individuals to exploit temporally and spatially variable resources, and has implications for population dynamics and population viability through the spatial redistribution of individuals [1]. Understanding the mechanisms that motivate individuals to disperse from, and to settle in specific locales is a fundamental question in ecology and conservation biology [1] particularly as populations are faced with increasing anthropogenic impacts and rapidly changing and fragmented environments. Animal dispersal is typically non-random, as informed dispersal decisions (i.e., those that are based on ecological or social cues) should confer an evolutionary advantage over uninformed decisions [2]. Dispersal behavior can thus be based on factors intrinsic to the individual (i.e., phenotype-dependent) and/or extrinsic social or ecological factors (i.e., condition-dependent) [3].

Numerous hypotheses have been proposed to explain the evolution of, and variation in dispersal behavior, yet most explanations involve competition, inbreeding avoidance, and/or environmental stochasticity [3], [4]. Individuals are theoretically expected to disperse from areas of high to low population density, to exploit areas with comparatively more abundant resources (e.g., space, food, mates) and areas with fewer competitors [3]. Results from the few empirical studies that have investigated density-dependent dispersal directly have found equivocal support for theoretical expectations, which may reflect context-specific probabilities of immigration and emigration in response to density [4]–[6]. Alternatively, when the relationship between population density and resource availability is not closely coupled, individuals may be better able to survive and reproduce by dispersing into locales characterized by habitat that is similar to habitat in their natal areas (i.e., natal habitat-biased dispersal [7], [8] or natal habitat imprinting [9], [10]).

Dispersal probability may also be inversely related to maternal age. Whether or not a female offspring disperses or exhibits philopatry is dependent upon the costs of dispersal as well as the costs of kin competition. Theory predicts that as a mother ages, her investment in future reproductive events (i.e., her residual reproductive value) declines [11]–[13]. Therefore, older mothers should invest more in the growth and survival of each current offspring (i.e., by promoting offspring philopatry) than younger mothers, who should invest more in their own growth and survival and thus future reproduction (i.e., by promoting offspring dispersal) [11], [13]. Dispersal is also commonly biased toward one sex, which reduces the risk of mating with close relatives, regardless of whether or not individuals are capable of recognizing and deliberately avoiding mating with kin [14]. In mammals, males are typically the dispersing sex while females remain philopatric [15], [16], yet there is variation among individuals of the same sex associated with phenotype and/or condition [17].

The above hypotheses are not mutually exclusive, and thus patterns in data, particularly when based on small sample sizes, may not characterize underlying processes unambiguously. Traditional studies of dispersal require marking and tracking a large number of individuals over time, and complications can arise due to difficulty obtaining adequate sample sizes and tracking long distance dispersers. Further, the limited duration over which observations are collected, particularly for long-lived, iteroparous species, can affect the accuracy and power of inferences [18]. For highly mobile vertebrates, long distance dispersers are difficult to track or recapture and differences in detectability between dispersers and non-dispersers may be large and could bias results [19]. Parentage analysis provides an alternative to traditional field-based methods (e.g., mark-recapture, radio telemetry), and has great potential to inform our understanding of dispersal [20], [21]. However, the logistical challenges associated with genetic sampling of a sufficient number of wide-ranging vertebrates have recently lead some researchers to question the feasibility of applying parentage analysis to investigate dispersal in these species [18].

**Figure 1:**
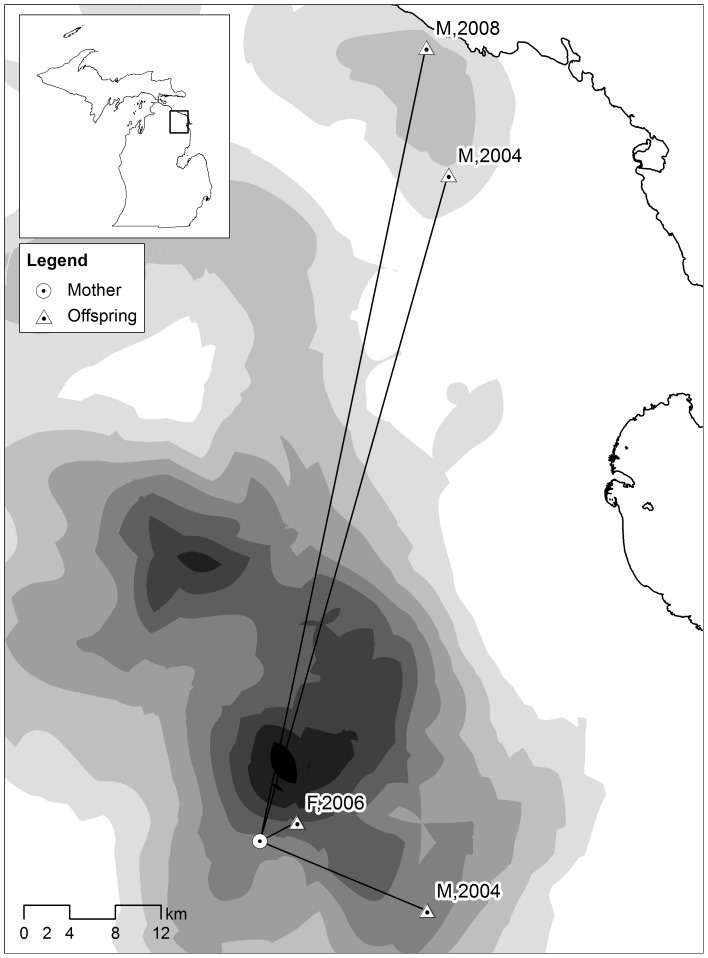
Example of black bear dispersal patterns. Example illustrating three cohorts of offspring from one black bear mother, showing patterns of sex-specific distances, and dispersal patterns in relation to harvest density (low  =  white, high  =  black). Offspring are labeled according to sex and year of birth. Square on inset indicates approximate area in Michigan.

In this study, we use a large number of harvest samples collected from an isolated, closed population of American black bears (*Ursus americanus*) inhabiting a large geographical area in the Northern Lower Peninsula (NLP) of Michigan, USA (Figure 1, inset). Black bears are solitary omnivores with a promiscuous mating system, male-biased dispersal [19], [21], and home ranges that can overlap considerably depending on resource availability [19], [22]–[24]. Annual population size estimates of NLP black bears over the last decade are stable and have ranged from 1,500–1,900 individuals, and local density varies. Between 13 and 29% of the population is harvested annually ([25]; unpublished data, Michigan Department of Natural Resources). Harvest quotas fluctuate annually, and are largely based upon the population estimates.

Parentage analysis using harvest samples has provided us with a unique opportunity to conduct a large-scale investigation of dispersal in a highly vagile species by quantifying sex-specific dispersal probabilities and distances and factors influencing these behaviors, like habitat quality and harvest density. We tested the following hypotheses:

1. Dispersal depends on sex and age of the offspring. Radio telemetry data indicate that dispersal in male NLP black bears usually has occurred by the time a bear reaches two years of age [26]. Thus, we predict that NLP black bears will follow the typical mammalian pattern of male-biased dispersal, and that the probability that a male has dispersed will increase after two years of age.
2. Female dispersal is inversely related to the age of the mother. We predict that females will be largely philopatric [16], and the mother will be increasingly more tolerant of offspring philopatry as she ages and her residual reproductive value declines.
3. Dispersal probability and dispersal distance are dependent upon harvest density. We predict that dispersal probability will be greatest in areas of high harvest density, where bears are more often displaced by hunting practices. Further, if harvest density is correlated with bear population density (e.g., for red grouse, [27]), we expect conspecific competition to be greatest at high density, thus increasing dispersal and causing dispersers to seek out areas with lower harvest density and lower bear density [28].
4. Black bears exhibit natal habitat-biased dispersal. Familiarity with a particular habitat type during formative early ontogenetic periods should increase an individual's ability to acquire resources in that habitat type. We predict that there will be a positive relationship between the habitat type at an individual's natal site and the habitat type into which that individual settles.

## Methods

### Study Area

Our study area covered the northern two-thirds of the lower peninsula of Michigan (∼47,739 km^2^). This area is considered an insular population, being bounded on three sides by the Great Lakes, and to the south by a landscape composed of intensive agricultural and expansive urban areas. The NLP landscape is a largely forested mix of northern and mixed hardwoods, pines, and forested and non-forested wetlands. The NLP is also highly fragmented by roads, forestry activities, and agriculture [29].

### Field Sampling

During the annual NLP bear hunting season (September and October), hunters must register harvested bears at registration stations facilitated by the Michigan Department of Natural Resources (MDNR). At the check stations, hunters report the bear's sex and harvest location (to a township, range, and section; 2.6 km^2^). A pre-molar tooth is extracted for aging and DNA extraction. Bears are aged by the MDNR using the cementum annuli method [30], which has been estimated to be 96% accurate (unpublished data, D. Etter). In seven years (2002 and 2003, and 2006–2010), MDNR personnel collected samples from 2,580 harvested bears. All samples were collected from bears that were legally harvested under bear hunting licenses issued by the MDNR to individual hunters. All harvest took place in one of three bear management units, and black bears are not threatened or endangered in the state of Michigan. No vertebrate Institutional Animal Care and Use Committee approval was sought because the harvest season takes place annually, it is strictly regulated by the MDNR, and no bear harvest was conducted specifically for the purpose of this study. Rather, harvest samples were provided by MDNR cooperators.

### Laboratory Analysis

We extracted DNA from bear teeth using Qiagen DNEasy Tissue Kits following manufacturer protocols (Qiagen Inc., Valencia, CA). DNA was quantified using a Nanodrop spectrophotometer (Thermo Scientific, Waltham, MA) and diluted to a 20 ng/μl working concentration. Using polymerase chain reaction (PCR), we amplified 12 microsatellite loci including *G10X*, *G10L*, *G10D*, *G10M*, *G10B* [31], *UarMU59*, *UarMU50* [32], *UT29*, *UT35*, *UT38* [33], *ABB1*, and *ABB4* [34]. 10 μl PCR reactions included 40 ng of DNA in 10 mM Tris-HCl, 50 mM KCl, 2.0 mM MgCl_2_, 0.2 mM of each dNTP, 1 pmol of each primer, and 0.5 units of *Taq* polymerase. Thermocycler conditions included a 4 min denaturation at 94°C, followed by 25–42 cycles of 30s at 94°C (1 min for *G10L*), 30s at the locus-specific annealing temperature (58°C for *G10X, G10L, G10D, G10B, UarMU59*, *UarMU59;* 54°C for *UT29, UT35, UT38, ABB1, ABB4*), 1 min at 72°C, and a final 10 min extension step at 72°C. We used 6.5% denaturing acrylamide gels for electrophoresis visualized on a LI-COR 4200 Global IR2 System (LI-COR Inc., Lincoln, NE). For each gel, we included molecular weight standards and individual bears with known genotypes. All alleles were scored independently by two experienced lab personnel using Saga genotyping software (LI-COR Inc., Lincoln, NE). 10% of samples were randomly selected and genotyped twice to provide a genotyping error rate of <2%.

Using MICRO-CHECKER [35], no loci were found to deviate significantly from Hardy-Weinberg and linkage equilibrium, so all 12 were retained for further analyses.

To minimize potential errors in field sexing, we genetically sexed 1) all females that were identified as having had cubs in consecutive years (female black bears typically produce young every other year [26]), 2) all female offspring that were identified as dispersers, and their mothers, and 3) all males that were identified as non-dispersers, and their mothers. Genetic sexing followed the protocol outlined in [36] using the *SE47* and *SE48* amelogenin primers [37].

### Parentage Analysis

Parentage analysis was conducted using the program FRANz [38] to identify mother-offspring (MO) dyads. FRANz is a Bayesian pedigree reconstruction program that allows for the incorporation of prior information about sex, birth year, and death year. Incorporating age priors of putative parents and offspring enables the program to handle multi-generational data, without *a priori* separation of individuals into cohorts. For each parent-offspring relationship identified, FRANz estimates a posterior probability of the identified parent being the true parent and a parent-pair log odds ratio (LOD) score [38], [39]. To assess the accuracy of parentage assignment and to set the threshold posterior probability for true parentage assignments, we first performed a simulation by assigning parentages to known (simulated) offspring (see File S1 for detailed methodology). In the simulation, FRANz identified the correct parents for 98.5% of the simulated offspring with posterior probabilities ranging from 0.41–1 (mean  = 0.96). We then performed a parentage analysis in FRANz using the real bear genotypes, and their sexes and birth and death years as priors, with the default parameter settings, with the exception of: maximum number of candidate fathers (Nmax)  = 800, our empirical estimate of genotyping error  = 0.02, the increment in steady state distribution variational distance (δ)  = 0.01, and the convergence tolerance (ε)  = 0.1. For further analyses, we retained only those MO dyads with posterior probabilities ≥0.9 (based on simulation results, see File S1).

### Spatial Analyses

Locations of mothers and offspring were georeferenced to the centroid of their reported harvest sections (a 2.6 km^2^ area). Euclidean distances between MO dyads were determined using ETGeowizards and Hawth's Tools [40] in ArcGIS 9.3 (ESRI, Redlands, CA). We classified offspring as ‘dispersers’ or ‘non-dispersers’ (hereafter called ‘residents’) based on threshold Euclidean distances between mother and offspring locations. Male dispersers were ≥30 km from the mother's location, and female dispersers were ≥20 km from the mother's location. Distance thresholds were set based on genetic spatial autocorrelation analyses that were performed separately for each sex (H. Draheim, unpublished data). Genetic spatial autocorrelation analyses examine the relatedness of pairs of bears at different distance classes, and can provide a good indicator of the extent of effective gene flow (i.e., dispersal) across the study area [41]. Results of the genetic spatial autocorrelation analyses showed that beyond the 20 and 30 km distance classes, female-female and male-male pairs, respectively, were no more related than expected by chance. We also assumed that beyond these distances, bears were outside of the core home ranges of assigned mothers [19], [22], [26], [29], [42].

To extract habitat information for each mother and offspring location, we reclassified the 2006 NOAA Coastal Change Analysis Program Land Cover dataset (resolution  = 30 m) into seven land cover classifications and ranked them according to bear habitat suitability (most (1) to least (5) suitable) based on an ecological model of bear habitat selection in Michigan (developed using radio telemetry and discrete-choice modeling [29]), as follows: mixed deciduous forest (MF, 1), forested wetland (FW, 1), evergreen forest (EF, 2), non-forested upland (NFU, 3), agriculture (AG, 4), non-forested wetland (NFW, 5), and developed (DEV, 5).

We determined localized harvest density using bear harvest locations. Although we do not have direct estimates of local population density, or harvest effort, we expect some degree of correlation between harvest density and bear density. One qualitative factor contributing to this supposition is that, in annual harvest surveys, hunters consistently cite bear density as the primary reason for selecting a hunting site [43]. Likewise, a previous study of NLP black bears found that the geographic distribution of harvest locations [44] (i.e., regions of relatively high and low harvest) does not show significant annual variation. As such, we used the harvest locations for each year from 2002–2010 to create annual kernel density function [45] grids and reclassified grids into categories ranging from 1–10 (low to high harvest density). We then created a median harvest density grid by calculating the median values over the nine annual harvest density grids. A 1.61 km diameter circular buffer (representing the approximate area of a section) was created around each harvest location, and we extracted harvest density and land cover grid cell values falling within each circular buffer. The value that constituted the majority of grid cells within a buffer was assigned for each individual's location.

### Statistical Analyses

We used mixed models (*Lme4* package in R [46]), to model the factors that were associated with dispersal probability (0, 1) and dispersal distance while accounting for repeated measures of mothers (random effect) with multiple offspring. We assumed that dispersal patterns did not change considerably over time, and thus pooled all data for analyses. To quantify associations between intrinsic and extrinsic factors affecting whether or not individuals disperse, we first modeled dispersal probability (with each offspring classified as a disperser (1) or resident (0), based on the distance thresholds presented above as a function of offspring sex (*offsex*), offspring age (*offage*), characteristics of the natal (mother's) site including habitat type (*momhab*), habitat suitability (*momhs*), and harvest density (*momharv*) and characteristics of the settled (offspring harvest) site including habitat type (*offhab*), habitat suitability (*offhs*), harvest density (*offharv*), and mother's age (at the time of offspring birth, *momage_corr*). Subsequently, using only those individuals classified as dispersers, we modeled dispersal distance as a function of offspring sex and age and characteristics of the settlement site (habitat type, habitat suitability, and harvest density) and the natal site (habitat type, habitat suitability, and harvest density). Lastly, again using only those individuals classified as dispersers, we investigated whether natal habitat-biased dispersal occurred. We modeled the habitat type at the settlement site as a function of offspring sex, offspring age, and the habitat type at the natal site. We tested 64 *a priori* hypothetical models of dispersal probability, 53 models of dispersal distance, and 7 models of habitat type (see File S2 for a complete list of models and results). We used Akaike's Information Criterion (AIC) for model selection, with the lowest values representing the best supported model(s). Once the top models were selected, a sex*age interaction was added to test for improved model fit. The sex*age interaction was aimed at capturing age-specific dispersal differences that may have only been present in one sex. Models were rescaled relative to the model with the lowest AIC value in the set (ΔAIC), and Akaike weights (*w_i_*) were calculated relative to the other models in each set. Models with ΔAIC values of ≤2 were considered to be supported.

## Results

We genotyped 2,540 black bears (1,415 males, 1,125 females) for inclusion in the parentage analysis to identify MO dyads. Parentage analysis identified 337 MO dyads with posterior probabilities ≥0.9. Dyads were comprised of 159 female and 178 male offspring, from 214 mothers. The average age at time of death for offspring, and at the time of offspring birth for mothers was 3.0 years (range  = 1–12), 2.5 years (range  = 1–14), and 5.8 years (range  = 2–22) for female offspring, male offspring, and mothers, respectively.

**Figure 2:**
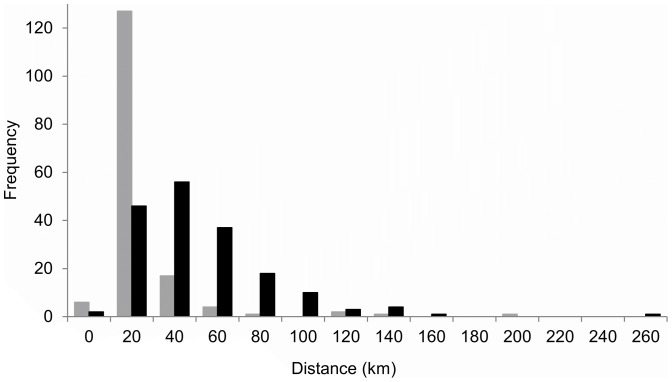
Distribution of black bear dispersal distances. Frequency histogram of pairwise distances between black bear mother and offspring dyads, for all individual males (black bars, n = 178) and females (grey bars, n = 159). Distances along the x-axis represent the upper bounds for each bin.

**Figure 3:**
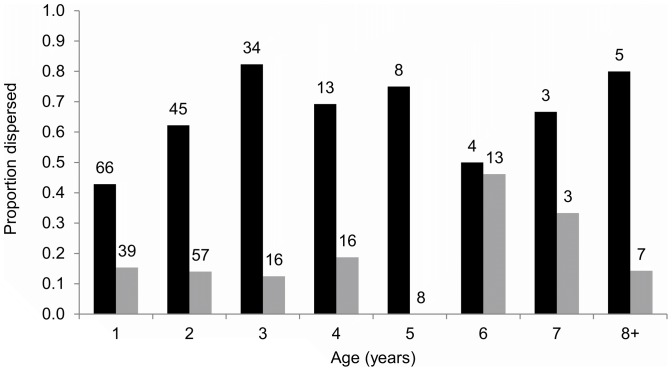
Dispersal probabilities, by age. Proportion of male (black bars) and female (grey bars) black bears that dispersed, by age. Sample sizes are presented above bars.

Euclidean distances averaged 14.2 km (median  = 7.7 km) between mother-female offspring dyads, and 41.1 km (median  = 34.9 km) for mother-male offspring dyads (Table 1, Figure 2). The maximum dispersal distance was 251.2 km for males and 187.2 km for females (Figure 2). 60.0% of male offspring and 17.0% of female offspring dispersed (Figure 3). For bears that were ≥3 years of age at the time of harvest (i.e., those that were most likely to have dispersed), the frequency of dispersal was 21% for females (n = 63) and 76% for males (n = 68). Dispersal distances for female dispersers averaged 48.9±7.7 km, while male dispersers averaged 59.0±3.2 km. Distances between mothers and female resident offspring averaged 7.1±0.4 km, while male resident offspring averaged 14.9±0.9 km (Table 1).

**Table 1:** Summary of pairwise Euclidean distances for black bear mother-offspring dyads, by offspring sex, including samples sizes (N), and ages of offspring (at time of death), and mothers (at time of offspring birth).

			Euclidean distance (km)		Offspring age (years)		Maternal age (years)	
		N	Mean (± SE)	Range	Mean (± SE)	Range	Mean (± SE)	Range
Males		178	41.1±2.5	0–251.2	2.5±0.2	1–14	5.8±0.3	2–19
	Dispersers	106	59.0±3.2	30–251.2	2.8±0.2	1–14	5.6±0.3	2–17
	Residents	72	14.9±0.9	0–28.8	2.1±0.2	1–11	6.0±0.5	2–19
Females		159	14.2±1.8	0–187.2	2.9±0.2	1–12	5.9±0.3	2–22
	Dispersers	26	48.9±7.7	20–187.2	3.5±0.5	1–9	4.9±0.7	2–13
	Residents	133	7.1±0.4	0–19.4	2.9±0.2	1–12	6.1±0.4	2–22

(Dispersers are defined by distances between natal and harvest locations (>30 km for males, and >20 km for females) based on results of an independent genetic spatial autocorrelation analysis)

The best model characterizing dispersal probability included offspring sex (*β_offsex_*  = 2.24), offspring age (*β_offage_*  = 0.18), and harvest density at the offspring settlement site (*β_offharv_*  = −0.39) (AIC  = 361.56; *w_i_*  = 0.22; Table 2, File S2). Competitive (ΔAIC<2) models of dispersal probability included harvest density at the natal site, as well as habitat suitability at both the natal and settlement sites (Table 2). Based on these models, males were more likely to disperse than females, and male dispersal probability increased considerably after 1–2 years of age (Figure 3). Dispersal probability was greatest for individuals dispersing from areas of high quality habitat and low harvest density and settling in areas of high quality habitat with low harvest density.

**Table 2:** Top ten linear mixed effects models of black bear dispersal probability and dispersal distance including Akaike's Information Criteria (AIC) rescaled to the lowest value (ΔAIC), and Akaike weights (*w_i_*).

Model parameters			
Response variable = dispersal probability	AIC	ΔAIC	*w_i_*
offsex + offage + offharv	361.6	0.0	0.22
offsex + offage + offharv + offhs	361.9	0.3	0.19
offsex + offage + offharv + momhs	362.8	1.2	0.12
offsex + offage + offharv + momhs + offhs	363.3	1.7	0.10
offsex + offage + momharv + offharv	363.5	2.0	0.08
offsex + offage + momharv + offharv + momage_corr	363.8	2.3	0.07
offsex + offage + momharv + offharv + offhs	363.9	2.3	0.07
offsex + offage + momharv + offharv + momhs	364.8	3.2	0.04
offsex + offage + momharv + offharv + momhs + offhs	365.2	3.7	0.04
offsex + offharv + offhs	367.9	6.3	0.01
**Response variable = dispersal distance**			
offsex * offage + offhab + momhab + offharv + momharv	1251.71	0.00	0.46
offsex * offage + offhab + momhab + offharv	1253.35	1.64	0.20
offsex + offage + offhab + momhab + offharv + momharv	1254.03	2.32	0.15
offsex * offage + offhab + momhab + momharv	1255.65	3.94	0.06
offsex + offage + offhab + momhab + offharv	1255.67	3.96	0.06
offsex * offage + offhab + momhab	1257.43	5.71	0.03
offsex + offage + offhab + momhab + momharv	1258.00	6.29	0.02
offsex + offage + offhab + momhab	1259.78	8.07	0.01
offsex + offhab + momhab	1261.64	9.93	0.00
offage + offhab + momhab	1286.34	34.63	0.00

Mother ID was included as a random effect in all models. Models with ΔAIC ≤ 2 are supported. See File S2 for a complete list of model results. (Model parameters are sex of offspring (*offsex*), age of offspring (*offage*), maternal age at the time of offspring birth (*momage_corr),* habitat type at the natal site (*momhab*), habitat type at the settlement site (*offhab*), harvest density at the natal site (*momharv*), harvest density at the settlement site (*offharv*), habitat suitability rank at the settlement site (*offhs*), and habitat suitability rank at the natal site (*momhs*), * indicates an interaction).

**Figure 4:**
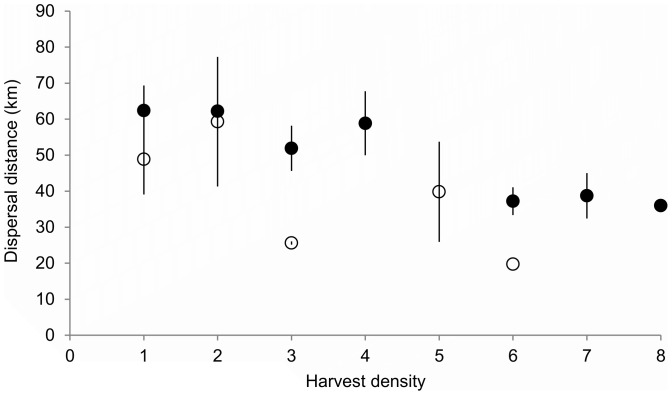
Dispersal distances and harvest densities. Average dispersal distances (±SE), based on harvest density at the settlement site (1–8, low to high, from rescaled kernel density function grid estimates of black bear harvest locations), for male (filled symbols) and female (open symbols) black bears.

The best model of dispersal distance included an interaction between offspring age and sex (β_offage*offsex_  = −0.7), harvest density (*β_momharv_*  = −1.5, *β_offharv_*  = −3.5) and habitat type at both the natal and the settlement sites (AIC  = 1251.7; *w_i_* = 0.46, Table 2, Figure 4). A competitive model (ΔAIC<2) included all of the factors in the best model with the exception of natal site harvest density. Dispersal distance was greatest for young males dispersing from areas with low harvest density and settling in areas with low harvest density (Figure 4). Generally, dispersal distances tended to be greater for bears dispersing from areas of lower quality habitat like non-forested upland (*β_NFU_*  = 7.52, mean distance  = 56.1 km) and non-forested wetland (*β_NFW_*  = 13.8, mean distance  = 62.1 km) and for individuals settling in more suitable habitat types like mixed forest (*β_MF_*  = −2.5, mean distance  = 53.9 km), forested wetland (*β_FW_*  = 2.1, mean distance  = 58.61 km), and evergreen forest (*β_EF_*  = −0.5, mean distance  = 63.35 km).

The best model of settled habitat type included offspring sex (*β_offsexM_*  = −0.2; AIC  = 357.3; *w_i_* = 0.66, File S2), and the next best model was a null model that included only the random effect of maternal identity. Thus, we found no evidence of natal habitat imprinting, but this could have been due more to the fact that most bears were found in one of two habitat types (mixed deciduous forest, 44%, and forested wetland, 38%), which may have limited the power to detect a biologically meaningful relationship. A higher proportion of female than male dispersers was harvested in mixed deciduous forest (59% of females vs. 39% of males), while a higher proportion of male than female dispersers was harvested in forested wetlands (26% of females vs. 41% of males).

The majority of dispersers either showed no change in harvest density between the natal versus the settled site (24%) or settled in a site with lower harvest density than in their natal site (44%) (see Figure 1 for an example). Only 33% of dispersers settled in an area with a higher harvest density than was observed in their natal site. Likewise, the majority of dispersers (70%, n = 95) showed no change in habitat suitability from the natal to settled site. Similar proportions of dispersers settled in sites with higher habitat suitability (13%) and lower habitat suitability (17%) compared to their natal site.

## Discussion

We have shown that parentage analysis is a powerful means of characterizing condition-dependent dispersal patterns in elusive species, particularly when data are collected over large temporal and spatial scales. We found that black bear dispersal in Michigan's NLP is strongly male biased with 76% of males, but only 21% of females dispersing. Furthermore, harvest density and habitat suitability had strong effects on dispersal probability and dispersal distances, and black bears did not appear to exhibit natal habitat-biased dispersal. The probability of dispersal increased with decreasing harvest density and increasing habitat quality at both the natal and settlement sites. Likewise, black bears in areas with low harvest density tended to travel greater distances, and most commonly settled in areas of high quality habitat.

Harvest pressure could be driving our observed pattern of higher dispersal probability and distance in areas with lower densities of bear harvest locations. Bears are primarily hunted in the NLP using bait and dogs [41]. If harvest density was driving our observed patterns of dispersal, we might expect to see higher dispersal in areas of high harvest density, due to competing bears being lured by bait stations, or regularly harassed by dogs. On the other hand, in areas of high harvest density, individuals who disperse greater distances may be more susceptible to harvest. Intensive harvest can result in reduced abundance [47], changes in offspring survival [48], skewed sex ratios [49], age distributions [47], [49], and distributions of phenotypic variation [50]. Anthropogenic selective pressures are imposed upon harvested populations that non-harvested populations are not subjected to [50], [51]. We speculate that high harvest intensity may either select for reduced dispersal or cause individuals to behaviorally modify their movement patterns (but see [52]).

Alternatively, black bear population density may be a strong regulator of dispersal in high density areas, via conspecific competition. Most dispersal hypotheses predict positive density-dependent dispersal resulting from intraspecific competition, which is strongest in territorial species and species that incur high dispersal costs [28], [53]. However, empirical studies, mostly based on small mammals [53]–[55], have shown that rates of immigration and emigration can also decrease at high density, due to saturation of aggressive and territorial individuals at high densities that restrict movements [5] of individuals into or out of these areas (i.e., the social fence hypothesis [56]). Black bears in areas of low density may not be limited by aggressive conspecifics, making them more able to move freely at lower cost [57], [58]. Using population-level spatial autocorrelation analysis, Roy et al. [58] showed that genetic spatial autocorrelation was strongest in low density black bear populations, which these authors attributed to restricted dispersal in high density populations [58]. Habitat quality was not found to be the strongest predictor of dispersal patterns for NLP bears. However, this may be due to the fact that much of the bear habitat in the NLP is high quality, so resources are not the strongest limiting factor.

Dispersal distances of NLP black bears are comparable to other studies of black and grizzly bears, where average female distances range from 2.9 to 14.3 km, and average male distances range from 34.0 to 61 km [21],[59],[60]. Long-distance dispersers (e.g., those individuals that make up the right-hand tails of the distributions in Figure 2) are rare in our study population. However, long-distance dispersers can be extremely important for population dynamics and gene flow, and the reasons for long- and short-distance dispersal may differ [17], [61]. Short-distance dispersal may be sufficient to avoid mating with kin, whereas long-distance dispersal may enable colonization of new areas, or escape from high density areas [17], [28]. Based on the variables measured, we were unable to identify any intrinsic characteristics (e.g., based on age, family, maternal age, birth year) that would differentiate long-distance dispersers from short-distance dispersers. Because of the importance of long-distance dispersal, further investigation into the factors that drive certain individuals to exhibit this behavior is warranted.

Approximately 24% of adult male black bears (≥3 years of age) remained within 30 km of their natal ranges. The relatively high percentage of non-dispersing male bears is surprising considering black bears are thought to unequivocally exhibit strong male-biased dispersal, with 100% of males dispersing in some study systems [21], [42], [59]. The NLP black bears experience high turnover of individuals that are removed by harvest (approximately 25% of the population is harvested annually [26]). In areas of high harvest density, juvenile males may be able to utilize habitats adjacent to their natal ranges that are left vacant following harvest of neighboring males. High male turnover would also contribute to our observed lower probability of dispersal in high harvest areas. Further investigation is needed to determine whether remaining near the natal range increases the likelihood of a male mating with kin [21], [62].

Comparisons of studies using ecological and genetic methods often reveal disparities in their results, even when conducted in the same study area. In a long-term radio telemetry study of NLP bears, Etter et al. (2002) found that 30 of 31 male bears (95%) dispersed, compared to the 52 of 68 male bears (76%) ≥3 years old that dispersed in our study (Fig. 3). The disparity in results may be attributed to differing methodology, data or sample sizes (i.e., differing thresholds defining dispersers vs. non-dispersers, different temporal span of the studies, order of magnitude difference in sample size), or the fact that the data from these two studies were collected a decade apart. Nevertheless, if differing methodology were the cause of the discrepancy, we would expect the telemetry data to provide an estimate of dispersal frequency that is biased low, due to the potential for dispersers to be lost from contact or incur higher mortality [18], [19]. Alternatively, harvest regimes and population density may have changed enough in a decade to impact dispersal frequency. In a study of black bears in New Mexico, Costello et al. [19], [21] found a similar pattern to our system, where almost all males tracked using radio telemetry dispersed [19], but a spatial analysis of genetic relatedness during the same time period was indicative of a much lower male dispersal rate (∼75%) [21].

Female philopatry, like male dispersal, is not absolute. Approximately 21% of females ≥3 years old emigrated, often over large distances (Figure 2). A similar result was found for NLP females using radio telemetry (9 of 28, or 32% of females dispersed [26]). The frequency of dispersal in female black bears in the NLP is, on average, 5–15% higher than estimates from previous studies of black bear populations [19], [23], [42], [63]. Relatively high female dispersal rates in our population may be indicative of overall low population density relative to other black bear populations, a low cost of dispersal for females, and/or abundant resources. Female black bears in the NLP are generally in good condition, which is reflected in a young age of first reproduction and high reproductive rates relative to other populations [26], [42]. Dispersal probability increased with the quality of the habitat, presumably because bears in better condition are less likely to incur major dispersal costs related to resource acquisition.

Although maternal age was not a strong predictor of offspring dispersal probability in our models, this might be due more to the small proportion of females that did disperse (i.e., limiting our statistical power) or to the intensive harvest that reduces the median age of mothers in the population. In Scandinavian brown bears, 41% of females disperse, and female dispersal probability is strongly negatively correlated with maternal age [62]. Brown bears form matrilineal assemblages, and female kin compete to remain in or near the home ranges of their mothers, which means that smaller female offspring are more likely to disperse [62]. For black bears in the NLP, maternal age was lower (by an average of one year) for female dispersers than for female residents (Table 1). This corresponds to our prediction that younger mothers should promote offspring dispersal in favor of investing more in their own growth and survival, which would enhance future reproductive efforts. We identified seven mothers who produced female offspring in multiple years and where one offspring dispersed and the others were philopatric. For all but one of these mothers, the disperser was the first born among her offspring. Harvesting mothers before they are able to reach an advanced age may confound what might otherwise be a strong tendency toward the formation of multi-generational matrilineal assemblages in black bears.

The putative alteration of natural dispersal patterns by intensive harvest is a situation deserving of management attention. Several studies comparing harvested and non-harvested bear populations have shown that vital rates, like reproductive success and survival, can be density-dependent and can be altered by intensive harvest [48], [64], [65]. Intensive harvest that reduces the probability of dispersal, regardless of the mechanism (behavioral plasticity or selection) could reduce population connectivity even if abundances are not declining. Our study provides evidence for a link between harvest density, population density, and dispersal probability that warrants further understanding for the management of harvested species.

## Supporting Information

File S1 **Methods and results of parentage analysis based on simulated black bear offspring.**
(DOCX)

File S2 **Full list of linear mixed effects models of black bear dispersal probability and dispersal distance.**
(XLSX)
