# Supplementary material for: Application of Large-Scale Parentage Analysis for Investigating Natal Dispersal in Highly Vagile Vertebrates: A Case Study of American Black Bears (Ursus americanus)
Source: PLoS One. 2014 Mar 12;9(3):e91168. doi: 10.1371/journal.pone.0091168 (PMC3951290; doi:10.1371/journal.pone.0091168)
Supplement: File S1 — Methods and results of parentage analysis based on simulated black bear offspring. (DOCX) [file pone.0091168.s001.docx]

**Supplementary File S1**. Methods and results of parentage simulation.

We used FRANz (Riester et al. 2009) to create a simulated dataset by randomly selecting two individuals from our data set and generating two offspring per parent pair by randomly selecting one allele from each parent at each locus. We created 500 parent pairs from which we generated 1,000 known offspring. Simulated offspring were randomly assigned a birth year, within the range of years in which the parents would have been alive together and capable of breeding. Death years were not assigned to simulated offspring. We included all simulated offspring (n = 1,000) and all bears in the real dataset from which the parents of the simulated offspring were drawn (n = 2,580) in the simulation FRANz analysis. We used the default parameter settings, with the exception of: Nmax = 800, typing error = 0.02, δ = 0.01, ε = 0.1; see Almudevar (2003) for a discussion of simulated annealing parameters used in pedigree reconstruction.

FRANz identified the correct parents for 98.5% of the simulated offspring with posterior probabilities ranging from 0.41 – 1 (mean = 0.96). Using these results, we set our threshold for acceptance of parentage assignments at 0.9. From the simulation, a threshold posterior probability of 0.9 would have caused us to reject 10.5% of true parentages (Type I error) while retaining only 0.6% of false parentages (Type II error). As we were most concerned with minimizing Type II error while still maximizing sample size, we felt that a threshold posterior probability of 0.9 was most appropriate.

**References**

Almudevar, A. 2003 A simulated annealing algorithm for maximum likelihood pedigree reconstruction. *Theor. Pop. Biol.* **63**(2), 63-75. (doi:10.1016/s0040-5809(02)00048-5).

Riester, M., Stadler, P. F. & Klemm, K. 2009 FRANz: reconstruction of wild multi-generation pedigrees. *Bioinformatics* **25**(16), 2134-2139. (doi:10.1093/bioinformatics/btp064).
